# Effect Produced by a Mixture of Phenol, *p*-Cresol, and Acetophenone on Four Species of Microalgae: Tolerance, Biodegradation, and Metabolic Alterations

**DOI:** 10.3390/toxics13100848

**Published:** 2025-10-06

**Authors:** Juan Manuel Rastrojo-Velasco, Rosa Leon, Ana Sayago, Angeles Fernandez-Recamales, Javier Vigara, Antonio Leon-Vaz

**Affiliations:** 1 Laboratory of Biochemistry, Faculty of Experimental Sciences, Marine International Campus of Excellence and REMSMA, University of Huelva, 21071 Huelva, Spain; juanmanuel.rastrojo@alu.uhu.es (J.M.R.-V.); rleon@uhu.es (R.L.); vigara@uhu.es (J.V.); 2 AgriFood Laboratory, Department of Chemistry, Faculty of Experimental Sciences, Campus de “El Carmen”, University of Huelva, 21071 Huelva, Spain; ana.sayago@dqcm.uhu.es (A.S.); recamales@uhu.es (A.F.-R.); 3 Institute of Sustainable Processes, University of Valladolid, Dr. Mergelina, s/n, 47011 Valladolid, Spain; 4 Department of Chemical Engineering and Environmental Technology, University of Valladolid, Dr. Mergelina, s/n, 47011 Valladolid, Spain

**Keywords:** acetophenone, ascorbate peroxidase, catalase, *Chlamydomonas reinhardtii*, *Chlorella sorokiniana*, *Nannochloropsis gaditana*, *p*-cresol, phenol hydroxylase, *Tetraselmis chuii*

## Abstract

Phenol-derived compounds are among the most hazardous organic pollutants for aquatic environments due to their relatively high aqueous solubility. Microalgae harbor metabolic pathways that enable the degradation of phenolic compounds into less toxic derivatives, highlighting their potential for the bioremediation of these contaminants. In this study, four microalgal species were evaluated for their tolerance and biodegradation capacity of a mixture of phenolic compounds which include phenol, *p*-cresol, and acetophenone. The results revealed that *Chlorella sorokiniana* and *Nannochloropsis gaditana* could tolerate higher concentrations of the mixture (60, 50, and 25 mg L^−1^ of phenol, *p*-cresol, and acetophenone) than *Chlamydomonas reinhardtii* and *Tetraselmis chuii* (60, 30, and 20 mg L^−1^ of phenol, *p*-cresol, and acetophenone). Notably, *Tetraselmis chuii* could biodegrade these compounds with the highest efficiency (32, 45, and 85% of initial phenol, *p*-cresol, and acetophenone, respectively) after 72 h of cultivation. In the absence of alternative carbon sources in the medium, *Tetraselmis chuii* also biodegraded 45, 60, and 51% of initial phenol, *p*-cresol, and acetophenone, at 72 h, highlighting its potential for bioremediation processes. Finally, the ascorbate peroxidase, catalase, and phenol hydroxylase enzymatic activities of *Tetraselmis chuii* were studied in presence of the pollutants, showing increasing activity levels of these enzymes (123, 135, and 173% of control cultures for APX, CAT, and PH, respectively) involved in the antioxidant system and the degradation of phenolic compounds.

## 1. Introduction

The pollution of aquatic environments produced by industrial wastewaters has been one of the main worldwide problems in these years. The growing demand for energy consumption and industrial products has contributed to the degradation of water quality in diverse ecosystems [1]. The presence of several pollutants, such as ammonium, phosphate, heavy metals, and organic compounds (pesticides, phenol derivatives, BTEXs), in industrial wastewaters is one of the main causes of this water quality degradation [2]. The increasing demand for diesel, petroleum, and plastic-derived products over the past century has promoted the petrochemical industry as one of the most important industries in the world. Industrial effluents originating from petrochemical industries contain high levels of phenolic compounds, aliphatic hydrocarbons, halogen-derived compounds, and mono- and polycyclic aromatic hydrocarbons (PAHs) [3]. Short-term exposure to these pollutants may cause different adverse effects, including pulmonary toxicity, immune responses, and thyroid destruction in humans and animals, as well as metabolic and DNA damage in microorganisms, even at low concentrations [4,5].

Phenol derivatives are one of the most abundant organic compounds in industrial and petrochemical effluents. They are spelled to the environment not only from petrochemical industries but also from leather, textile, and pharmaceuticals. Their ubiquity and acceptable solubility in water, compared to PAHs or aliphatic hydrocarbons, make them one of the most problematic organic pollutants [6]. The removal of phenolic compounds has been performed with physical and chemical methods, such as adsorption, flocculation, and oxidation [7,8]. However, these approaches have serious problems with their operational costs at low concentrations of these compounds [9].

The microbial degradation of these pollutants has emerged as a promising approach to addressing this issue in aquatic environments. Microalgae are a group of photosynthetic organisms able to grow under different stress conditions, including heavy metals and both organic and inorganic contaminants [10,11,12]. Some freshwater microalgae, such as *Chlamydomonas reinhardtii* and *Chlorella sorokiniana*, have been described to use phenol derivatives compounds as alternative carbon sources [13,14]. Moreover, the increasing interest in marine microalgal strains is related to the high salinity typically found in petrochemical and phenol-derived wastewaters [15]. In this context, marine microalgae with rigid cell walls, such as *Nannochloropsis gaditana* and *Tetraselmis chuii*, represent promising candidates, as they have previously demonstrated their capacity to remove phenol derivative compounds from wastewaters [16,17]. Different microalgae have been tested for the biodegradation of phenolic compounds, with a wide range of results. While most microalgae can remove phenol or its derivatives when present as single pollutant in the culture medium, their efficiency decreases when there is a mix of different compounds [17,18]. These limitations in the simultaneous biodegradation of more than one pollutant in the culture medium may be caused by additive or synergetic toxicity effect, which reduces the microalgal biodegradation capacity [19]. However, due to the lack of studies involving mixtures of phenol derivative compounds, the underlying mechanisms still remain unclear. Thus, further research is needed to evaluate microalgal responses to pollutant mixtures.

The presence of phenolic compounds in the culture medium of microalgae can also produce alterations in microalgae metabolism. Previous studies have reported an increase in reactive oxygen species (ROS) production and, consequently, an induction of the antioxidant system, including catalase (CAT), ascorbate peroxidase (APX), and superoxide dismutase (SOD) activity, as well as an increase in lipid content [18,20,21]. Additionally, enzymes involved in the biodegradation of these compounds, such as phenol hydroxylase (PH) and catechol 2,3-dioxygenase, are also induced under these stress conditions [13,22,23]. Thus, obtaining a better comprehension of the effect that phenol derivative compounds produce on microalgae metabolism could be a first step for the improvement of pollutant biodegradation. However, to date, there are just a few reports on the removal of phenolic compounds in a mixture of three compounds. In this work, four species of microalgae were tested to elucidate their tolerance and biodegradation capacity for a mixture of pollutants that includes phenol, *p*-cresol, and acetophenone. The microalgal strains were *Chlamydomonas reinhardtii*, *Chlorella sorokiniana*, *Tetraselmis chuii*, and *Nannochloropsis gaditana*, which were cultured with concentrations of 60 mg L^−1^ of phenol, 30 or 50 mg L^−1^ of *p*-cresol, and 20 or 25 mg L^−1^ of acetophenone, which are typical concentrations of these compounds after secondary wastewater treatments in phenol production plants [6,24]. Additionally, the enzymatic activity of the enzymes PH (EC:1.14.13.7), APX (EC:1.11.1.11), and CAT (EC:1.11.1.6) were studied after 72 h of culture under phenolic compound stress in the microalga *T. chuii* to check how these compounds alter the metabolism of this strain.

## 2. Materials and Methods

### 2.1. Microalgae Strain and Culture Conditions

Microalgae used in this work were the freshwater species *Chlamydomonas reinhardtii cc-1690 WTmt*-21gr and *Chlorella sorokiniana* 211-32, kindly provided by the University of Córdoba and the Institute of Plant Biochemistry and Photosynthesis (IVBF), respectively, and the seawater species *Tetraselmis chuii* CCMM 03/0201 and *Nannochloropsis gaditana* CCMM 04/0201 from the culture collection of the Institute of Marine Sciences of Andalusia (ICMAN). Freshwater strains were cultured in Tris-Acetate-Phosphate (TAP) medium [25], while marine strains were cultured in Guillard F/2 medium [26] using CO_2_ as carbon source with continuous aeration (3% CO_2_). Microalgal pre-inocula were added to the medium at an initial optical density OD_660_ of 1.2. Phenolic compounds were added to the culture medium after autoclaving at the concentrations described in Table 1, and pH was adjusted to 6.7. The microalgae were cultured at 25 ˚C under continuous agitation (120 rpm) and light irradiation (120 µmol m^−2^ s^−1^).

### 2.2. Biodegradation Experiments

The four microalgal strains were cultured as indicated in 2.1. For cultures without a carbon source, pre-inocula were harvested and resuspended in fresh medium without carbon source at an initial optical density OD_660_ = 1.2. TAP medium was prepared without acetate, the pH was adjusted to 6.7 before autoclaving, and F/2 strains were cultured without CO_2_ aeration. The high cell density prevented the occurrence of a lag phase in the cultures and provided a protective effect against the toxicity of the tested pollutants, and samples were taken at 24 and 72 h of culture to determine the pollutant concentration. These 2 mL aliquots were centrifuged at 13,400× *g*, the supernatants were filtered using 0.22 nylon filters, and the samples were stored at −20 °C for subsequent phenolic compound determination.

### 2.3. Determination of Phenolic Compounds

Phenolic compounds were determined by HPLC using an Agilent 1100 series system (Agilent, Santa Clara, CA, USA) equipped with a binary pump system, vacuum degasser, a thermos stated column, and a diode array detector (DAD). Separation of phenolic compounds was performed in a Nucleosil 100C18 (4.6 mm × 250 mm, 5 μm) analytical column. For the successful resolution of the compounds, the elution program was performed using an isocratic flow, with two mobile phases at a flow rate of 1.2 mL min^−1^ and a temperature of 40 °C. Solvents were acetonitrile (A) and water (B) in a proportion of 55:45. The injection volume was 20 μL, and the program was recording the absorbance for 12 min until the three peaks were detected. Chromatograms were recorded at 271 and 290 nm. Retention times for the compounds were 2.92 min for phenol, 4.89 min for *p*-cresol, and 6.34 min for acetophenone. Quantification was carried out by comparing the areas with the calibration standards (from 1 to 100 mg L^−1^ of each compound).

### 2.4. Enzymatic Assay Experiments

Enzymatic assays were performed using crude extract of the microalga *T. chuii*. For the extraction of soluble proteins, samples were taken from the control and phenol derivatives-treated cultures at 72 h. These aliquots were centrifuged at 4400× *g*, and the supernatants were discarded. The wet biomass was resuspended in 50 mM phosphate buffer (pH 7.5) and disrupted using glass beads in a Digital Disruptor Genie^®^ (Scientific Industries, Bohemia, NY, USA) for 3 cycles of 30 s. After that, the homogenate was centrifuged at 14,000× *g* for 15 min, and the supernatants were used as crude extract source. The Bio-Rad Bradford assay was used to determine protein in a crude extract according to the manufacture’s protocol, using BSA as the standard.

APX and CAT enzymatic activity were determined kinetically as previously described by Romero-Cruz et al. [10]. One unit of APX was defined as the amount of enzyme that oxidizes 1 µmol of ascorbate min^−1^. One unit of CAT was defined as the amount of enzyme required to decompose 1 µmol of H_2_O_2_ min^−1^. PH activity assay was determined spectrophotometrically by measuring the NADPH content at 340 nm. The kinetics of NADPH disappearance was analyzed as described by Wang et al., [27] with minor modifications. The measured mixture contained, in a final volume of 1 mL, 50 mM Tris-HCl (pH 7.5), 5 μM NADPH, 5 nM FAD, 60 μM phenol, and 100 μL of crude extract. One unit of PH was defined as the amount of enzyme required to oxidize 1 μmol of NADPH min^−1^.

### 2.5. Statistical Analysis

All the experiments were carried out using biological triplicates and represented as mean value ± SD. One-way analysis of variance (ANOVA) was applied to identify significant differences between conditions. Significant differences were considered for values with *p* < 0.05 and *p* < 0.01. Statistical analyses were performed using IBM SPSS Statistics v29.0 software (Armonk, New York, NY, USA).

## 3. Results and Discussion

### 3.1. Tolerance of Microalgae to a Mixture of Phenol-Derived Compounds

**Figure 1:**
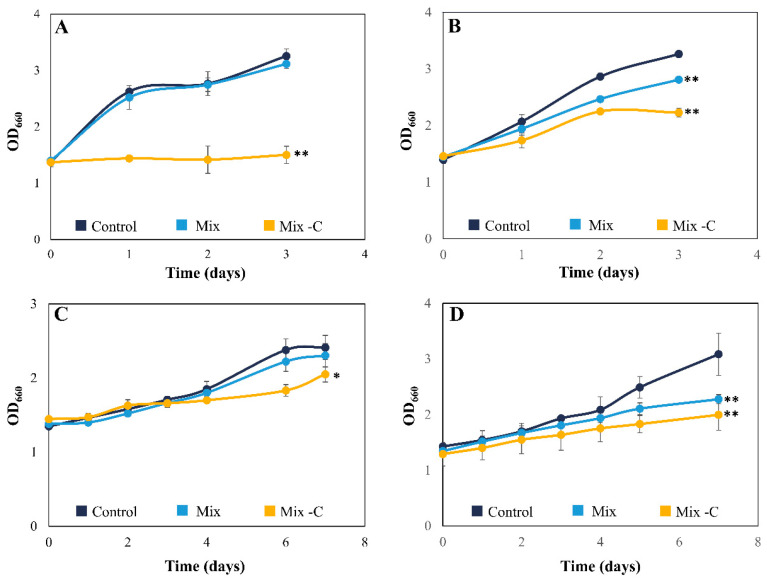
Growth curves expressed as mean ± SD of *C. reinhardtii* (**A**), *C. sorokiniana* (**B**), *T. chuii* (**C**), and *N. gaditana* (**D**) under a mixture of phenol, *p*-cresol, and acetophenone cultured with the standard medium (blue line) and without the carbon source of the medium (yellow line). Initial concentrations of the phenol derivate compounds were 60 mg L^−1^ of phenol, 30 mg L^−1^ of *p*-cresol, and 20 mg L^−1^ of acetophenone for *C. reinhardtii* and *T. chuii*; 60 mg L^−1^ of phenol, 50 mg L^−1^ of *p*-cresol, and 25 mg L^−1^ of acetophenone for *C. sorokiniana* and *N. gaditana*. * Significant differences in biomass between control and mixture of pollutants treatment at *p* < 0.05 and ** at *p* < 0.01.

The mix of phenolic compounds was tested using two different concentrations, depending on the tolerance of each species. While *Chlorella sorokiniana* (*C. sorokiniana*) and *Nannochloropsis gaditana* (*N. gaditana*) tolerated higher concentrations of *p*-cresol and acetophenone (50 and 25 mg L^−1^, respectively), *Chlamydomonas reinhardtii* (*C. reinhardtii*) and *Tetraselmis chuii* (*T. chuii*) could not grow under these conditions. Thus, the studies with these two strains were performed using a mixture of phenol derivate compounds with lower concentrations of *p*-cresol and acetophenone (30 and 20 mg L^−1^, respectively), as described in Table 1. Exposure to the mixture led to a slight decrease in *C. reinhardtii* and *T. chuii* growth, whose biomass decreased only 5% compared to control cultures (Figure 1A,C). In contrast, there was a significant (*p* < 0.01) reduction in *C. sorokiniana* and *N. gaditana* biomass, with 15 and 27% less biomass than in control cultures, respectively (Figure 1B,D). It is noteworthy that *C. sorokiniana* and *N. gaditana* mix cultures had similar growth curves to the control at the beginning of the experiment, with the differences emerging at the mid-exponential phase (2 and 5 days, respectively). This change in *C. sorokiniana* can be related to metabolic modifications. Previous studies demonstrated that *C. sorokiniana* can assimilate the initial concentration of acetate in TAP medium after 24–48 h [28]; consequently, the microalga could use the tested pollutants as a carbon source once acetate was depleted. There are also numerous studies which have reported that different microalgae can grow at phenol concentrations up to 200 mg L^−1^, demonstrating the high tolerance of certain species to this organic compound [22,29,30]. However, only a few studies have tackled the tolerance and cometabolic biodegradation of phenol and *o/p*-cresol, and these have consistently shown reduced tolerance when microalgae were exposed to a mixture of pollutants rather than individual compounds [17,18]. These results may explain the low tolerance of the microalgae tested in this work compared with previous studies using a single pollutant [13,17].

The four microalgal strains were also exposed to the mix of phenol derivate compounds in the absence of another carbon source in the medium (acetate or CO_2_ aeration) (-C). Under these stress conditions, the strains could adapt their metabolism to use these compounds as alternative carbon source, as previously described [13]. The results shown in Figure 1 for -C conditions were diverse. On the one hand, *C. reinhardtii* was not able to grow, obtaining similar biomass values throughout the experiment (Figure 1A). On the other hand, *C. sorokiniana* and *N. gaditana* showed a significant decrease (*p* < 0.01) compared to the control, with 32 and 38% less biomass, respectively (Figure 1B,D). *T. chuii* was the only species whose growth curve was similar to the control, although there were minor differences at the end of the exponential phase (Figure 1C). These results confirm that *C. sorokiniana*, *T. chuii*, and *N. gaditana* can use phenol derivative compounds as a carbon source for their development. Microalgae usually need an adaptation period to modify their metabolism in order to change their preferred carbon source [22]; however, *T. chuii* was able to perform this metabolic adjustment without this period, as Figure 1C shows. These results are consistent with the rapid adaptation to oxidative microenvironments that *T. chuii* showed through alterations in its transcriptome and metabolome [31]. Although most of the studies were carried out with freshwater species, the interest of the genera *Tetraselmis* for removing phenolic compounds is increasing in recent years [17,32,33]. The use of seawater species, such as *T. chuii*, has also the advantage that these species are more adapted to the high salinity typically found in industrial wastewaters (up to 100 g L^−1^ of salts) [15,34].

### 3.2. Biodegradation of Phenolic Compounds

**Figure 2:**
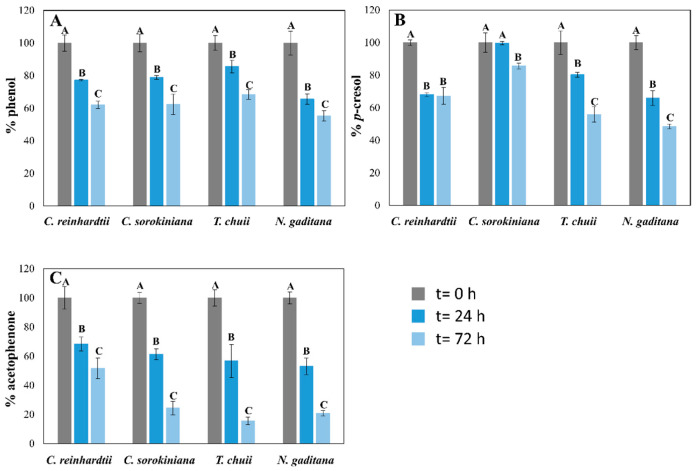
Biodegradation of phenol (**A**), *p*-cresol (**B**), and acetophenone (**C**), expressed as mean ± SD, after 24 and 72 h of cultured in the mixture with the standard medium (filled) cultures of the four microalgae tested. Initial concentrations of the phenol derivate compounds were 60 mg L^−1^ of phenol, 30 mg L^−1^ of *p*-cresol, and 20 mg L^−1^ of acetophenone for *C. reinhardtii* and *T. chuii*; 60 mg L^−1^ of phenol, 50 mg L^−1^ of *p*-cresol, and 25 mg L^−1^ of acetophenone for *C. sorokiniana* and *N. gaditana*. For the same microalgae, different capital letters indicate statistically significant differences (*p* < 0.05) between timepoints.

**Figure 3:**
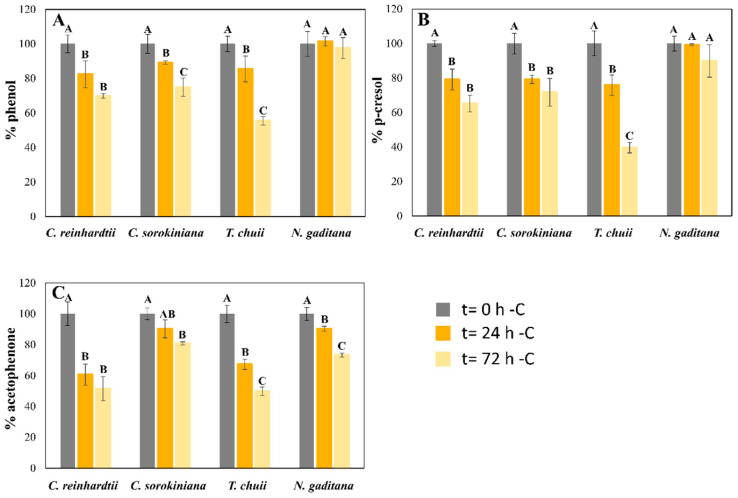
Biodegradation of phenol (**A**), *p*-cresol (**B**), and acetophenone (**C**), expressed as mean ± SD after 24 and 72 h of cultured in the mixture, with and without a carbon source, in cultures of the four microalgae tested. Initial concentrations of the phenol derivate compounds were 60 mg L^−1^ of phenol, 30 mg L^−1^ of *p*-cresol, and 20 mg L^−1^ of acetophenone for *C. reinhardtii* and *T. chuii*; 60 mg L^−1^ of phenol, 50 mg L^−1^ of *p*-cresol, and 25 mg L^−1^ of acetophenone for *C. sorokiniana* and *N. gaditana*. For the same microalgae, different capital letters indicate statistically significant differences (*p* < 0.05) between timepoints.

The capacity of the four microalgal strains to degrade phenol, *p*-cresol, and acetophenone from the mixture was also reported at 24 and 72 h of culture. Additionally, a control without microalgae was included, demonstrating that the concentrations of the pollutants remained stable after 72 h (losses of less than 2.5%). The results demonstrate that these four microalgae could biodegrade these pollutants from the culture medium (Figure 2 and Figure 3). The biodegradation levels of phenol were slightly higher in seawater than in freshwater microalgae in the standard experiment (Figure 2). While *T. chuii* and *N. gaditana* degraded 32 and 45% of the initial phenol, respectively, *C. reinhardtii* and *C. sorokiniana* biodegraded 38 and 39%, after 72 h (Figure 2A). These results changed in the -C cultures (Figure 3). In this experiment, the removal values in freshwater species were lower than with a carbon source (31 and 25% for *C. reinhardtii* and *C. sorokiniana*, respectively), after 72 h. However, *T. chuii* was able to biodegrade 45% of the total phenol from the culture medium in the same period (Figure 3A), demonstrating its rapid metabolic shift from phototrophic to mixotrophic growth using phenol as a carbon source in a short period of time. Different studies have reported the capacity of the genus *Tetraselmis* to rapidly adapt to stress conditions [31,35], which may underline its capacity to efficiently assimilate these pollutants. *N. gaditana* suffered a significant decrease in phenol biodegradation levels in these conditions compared with standard cultivation (Figure 2A), keeping similar concentrations of this pollutant than the initial ones after 72 h (Figure 3A). Although there was no cell growth in *C. reinhardtii* -C cultures, as Figure 1A shows, some phenol removal was observed (Figure 1A), which means that phenol decrease can be mainly produced by cell surface adsorption instead of biodegradation. Though the mixture of pollutants seems to be toxic for this microalga, Nazos et al. [13] reported that *C. reinhardtii* could biodegrade phenol as a single carbon source, showing also a production of catechol in the medium. Biodegradation results of phenol in the four microalgae tested were lower than previous studies using this pollutant as a single one (Table 2) [11,18,30], which confirms that mixtures of pollutants, such as those used in our work, are more difficult to remove. These results are consistent with findings by Meza-Escalante et al. [17], which demonstrated that in a mixture of phenol derivate compounds (phenol, *o*- and *p*-cresol), biodegradation of phenol decrease significantly compared with its degradation as a single pollutant, being the biodegradation capacity of *o/p*-cresol higher than phenol (Table 2), in concordance with the results reported in Figure 2 of our work.

The biodegradation of *p*-cresol reported similar behaviors than phenol. The removal values were from 15% in *C. sorokiniana* cultures to 52% in *N. gaditana* cultures in the carbon source supplied (+C) experiment after three days (Figure 2B). Although *C. sorokiniana* tolerated high concentrations of this pollutant, it exhibited limited biodegradation in the mix with other pollutants, being the affinity for phenol and acetophenone higher than *p*-cresol under +C conditions (Figure 2). However, under -C conditions, different behaviors were reported for the four microalgae (Figure 3B). While *N. gaditana* could not biodegrade *p*-cresol in -C conditions, *T. chuii* and *C. sorokiniana* had higher biodegradation levels than in +C conditions (60 vs. 45% and 29 vs. 15%, respectively) after 72 h (Figure 2B and Figure 3B). Finally, *C. reinhardtii* could adsorb 35% of initial *p*-cresol in -C conditions, as happened with phenol (Figure 2A and Figure 3A). The removal of carbon source (-C) also produced an increase in *T. chuii* degradation rates from 45% to 60% of the initial *p*-cresol concentration (Figure 2B and Figure 3B). These results are in agreement with previous works showing that *Tetraselmis suecica* degrades *o*- and *p*-cresol more efficiently than phenol in a mix of pollutants [17]. Xiao et al. [18] also tested the microalga *Chlorella vulgaris* in the presence of different concentrations of phenol and *p*-cresol, demonstrating that low concentrations of phenol in the culture medium promoted the biodegradation of *p*-cresol in microalgae (Table 2).

Acetophenone biodegradation exhibited similar patterns in three of the studied microalgae, where biodegradation levels in +C conditions were higher than in -C. *C. reinhardtii* was the only strain which removed similar concentrations in both conditions (Figure 2C and Figure 3C), as for the other pollutants (Figure 2 and Figure 3), probably as an adsorption process in the -C experiment due to the lack of cellular growth (Figure 1A). Nevertheless, *C. sorokiniana, T. chuii*, and *N.gaditana* could remove 75, 85, and 80%, respectively, of initial acetophenone in the +C cultures after 72 h. These removal rates decreased in -C experiments until 20, 50, and 25% of initial concentration (Figure 2C), probably due to its higher metabolic complexity in degradation pathway compared with phenol and *p*-cresol [36]. Although it is more toxic to microalgae than the other pollutants tested, acetophenone was the least studied of the proposed mix in microalgae. The lack of studies in literature limits direct comparisons with previous research. The microalga *Tribonema* sp. could remove high amounts of this pollutant using catalytic intense pulse light as a activating (Table 2) [20]. Nevertheless, the contribution of the microalga cannot be clearly established. Thus, acetophenone could produce more alterations in microalgae metabolism, being its biodegradation a priority for microalgae development, which could explain the results shown in Figure 2. However, their preference could change when the carbon source is removed, and phenol can be the most adequate nutrient for them because of its biodegradation pathway, which makes this pollutant easier than the others to be assimilated by microalgae [36].

### 3.3. Effect of Phenolic Compounds on the Activity Level of Antioxidant Enzymes in Different Microalgae

**Figure 4:**
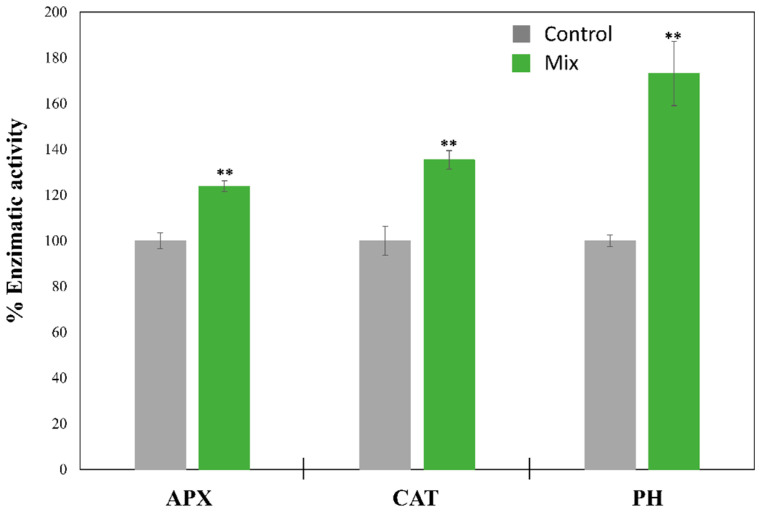
Effect of a mixture of phenol, *p*-cresol, and acetophenone on the enzymatic activities of APX, CAT, and PH, expressed as mean ± SD in *Tetraselmis chuii* after 72 h of cultivation. Initial concentrations of the phenol derivate compounds were 60 mg L^−1^ of phenol, 30 mg L^−1^ of *p*-cresol, and 20 mg L^−1^ of acetophenone. 100% APX, CAT, and PH activity corresponded to 4.428 ± 0.153 U mg^−1^, 10.073 ± 0.648 U mg^−1^, and 296.611 ± 7.535 U mg^−1^, respectively. ** Significant differences in biomass between control and mixture of pollutant treatment at *p* < 0.01.

The presence of these pollutants in the culture medium could produce alterations in microalgal metabolism, increasing the concentration of ROS species and activating the antioxidant system of the microorganisms [3]. Furthermore, some enzymes that can biodegrade these compounds are also expressed under these stress conditions [22]. Thus, the enzymatic activity of ascorbate peroxidase (APX), catalase (CAT), and phenol hydroxylase (PH) was studied in the marine microalga *T. chuii*, as selected model due to its high capacity to biodegrade the selected pollutants under both standard and -C conditions, after 72 of cultivation under phenol derivative stress. The results, represented in Figure 4, showed a significant (*p* < 0.01) increase in antioxidant enzymatic activities with APX and CAT activities of 1.24 and 1.35 times higher than control cultures, respectively. These results after that period of exposure are expected due to the harmful effects of these pollutants in microorganisms. It has been demonstrated that CAT plays a crucial role in the elimination of ROS species under phenol stress in microalgae. In this context, a transcriptomic study performed by Zhou et al. [37] demonstrated that three different genes related with CAT synthesis were upregulated from four to seven times in the microalga *Chlorella* sp. L5 after 96 h of exposure to 500 mg L^−1^ of phenol. Moreover, other studies reported increases from 1.3 to 4 times in CAT enzymatic activity due to the presence of phenol (from 50 to 500 mg L^−1^) in the microalgal species *Chlorella* sp., *Lingulodinium polyedrum*, and *Scenedesmus abundans* [23,38,39], which are in agreement with the results obtained for *T. chuii* in this work. However, the role of APX in microalgae under phenol stress is more variable. While Zhou et al. [37] reported an upregulation of different genes related to this enzyme at high concentrations of phenol (500 mg L^−1^) in *Chlorella* sp. L5, other studies showed no significant differences in the microalga *Lingulodinium polyedrum* with a phenol concentration of 9.5 mg L^−1^, or a significant decrease in APX activity at concentrations from 50 to 300 mg L^−1^ in the microalga *Scenedesmus abundans* [23,39]. In contrast, a recent study performed by Lu et al. [40] reported an increase in monodehydroascorbate (MDA) production due to a *p*-cresol derivative in the microalga *Phaeodactylum tricornutm*, being MDA a direct product of APX activity. These results suggest that the increase in APX reported by *T. chuii* in our work may be related to *p*-cresol exposure. Moreover, APX activity tends to decrease at high pollutant concentrations due to enzyme inactivation or depletion of ascorbate, as was described by Romero-Cruz et al. under Cu^2+^, Cd^2+^, Hg^2+^, and As (III) stress [10].

The enzymatic activity of *T. chuii* PH was also significantly (*p* < 0.01) enhanced in the presence of a mixture of phenol derivate compounds, with an increase of 1.73 times compared with the control cultures activity (Figure 4). This enzyme, which is responsible of phenol oxidation to 2,3-chatecol, constitutes the first step of the phenol biodegradation pathway, which ends in the production of acetyl CoA or pyruvate [13]. Moreover, phenol is a secondary metabolite in *p*-cresol and acetophenone biodegradation, which is produced through oxidation [20,41]. Thus, these results in PH enzymatic activity confirm that *T. chuii* is able to biodegrade phenol into catechol, introducing this compound into the carbon assimilation pathway, as was previously described for other microalgal strains such as *Chlorella vulgaris* and *Chlamydomonas reinhardtii* [11,13]. Similar increases in PH activity (1.63 times) was previously reported in the marine microalga *Isochrysis galbana* at 50 mg L^−1^ of phenol [27]. However, the enzymatic activity of PH was inhibited at higher concentrations of phenol in this study, just as their biodegradation rates. Furthermore, it has been reported that the cometabolic degradation of phenol and *p*-cresol increased the PH activity in the microalga *C. vulgaris* [18], which is in concordance with the results obtained for *T. chuii* in this work (Figure 4). Thus, although PH activity is an outstanding first approach to understanding the mechanisms that *T. chuii* uses to biodegrade these pollutants, further research focusing on molecular or omics approaches is needed for a full comprehension of *T. chuii* metabolism under phenol derivative stress.

## 4. Conclusions

In this study, the high tolerance of different microalgae to a mixture of phenolic compounds has been demonstrated, *Chlorella sorokiniana* and *Nannochloropsis gaditana* being the most tolerant strains. Moreover, the capacity of these microalgae for the biodegradation of phenolic compounds was also tested in the four strains. In this case, *Tetraselmis chuii* showed higher biodegradation capacity, both with and without a carbon source in the medium. The metabolic alterations produced by these compounds in *Tetraselmis chuii* were also studied, revealing an enhancement of the antioxidant system through APX and CAT activities as well as the capacity to biodegrade these compounds with an increase in PH enzymatic activity. These results confirmed that *Tetraselmis chuii* could be an outstanding tool for the biodegradation of phenol derivative pollutants spilled in different industry wastewaters, due to its robustness, biodegradation capacity in the absence of a carbon source, and tolerance to high salinity conditions, thereby mitigating the impact of these effluents on aquatic and marine ecosystems.

## Figures and Tables

**Table 1:** Initial concentration of pollutants tested.

Strain	Initial Concentration (mg L^−1^)		
	Phenol	*p*-cresol	Acetophenone
*C. reinhardtii*	60	30	20
*C. sorokiniana*	60	50	25
*T. chuii*	60	30	20
*N. gaditana*	60	50	25

**Table 2:** Comparison of biodegradation of phenolic compounds in different microalgae.

Pollutant	Organism	Initial Concentration	Degradation	Time	Reference
Phenol	*C. reinhardtii*	375 mg L^−1^	23%	240 h	[13]
	*Tetraselmis suecica*	100 mg L^−1^	90%	192 h	[17]
	*Chlorella vulgaris*	100 mg L^−1^	100%	96 h	[18]
		300 mg L^−1^	58%		
	*Chlorella pyrenoidosa*	200 mg L^−1^	100%	144 h	[30]
	*Chlorella vulgaris*	100 mg L^−1^	100%	144 h	[11]
Acetophenone	*Tribonema* sp. + Catalytic intense pulse light	100 mg L^−1^	80%	0.7 h	[20]
*p*-cresol	*Tetraselmis suecica*	100 mg L^−1^	85%	192 h	[17]
	*Chlorella vulgaris*	100 mg L^−1^	100%	96 h	[18]
		300 mg L^−1^	40%		
Mixture	*Tetraselmis suecica*			168 h	[17]
Phenol		40 mg L^−1^	12%		
*o*-cresol		35 mg L^−1^	29%		
*p*-cresol		35 mg L^−1^	74%		
Mixture	*Chlorella vulgaris*			96 h	[18]
Phenol		100 mg L^−1^	57%		
*p*-cresol		300 mg L^−1^	55%		
Mixture	*T. chuii*			72 h	This study
Phenol		60 mg L^−1^	32%		
*p*-cresol		30 mg L^−1^	45%		
Acetophenone		20 mg L^−1^	85%		

## Data Availability

The original contributions presented in the study are included in the article, further inquiries can be directed to the corresponding author.

## Abbreviations

The following abbreviations are used in this manuscript:

APX	Ascorbate peroxidase
CAT	Catalase
PH	Phenol hydroxylase
